# Automated CEAP classification of venous duplex reports using multimodal artificial intelligence

**DOI:** 10.1016/j.jvsv.2026.102613

**Published:** 2026-09-01

**Authors:** Joseph Cutteridge, Henry Bergman, Will Jackson, Manj Gohel, Alun Davies

**Affiliations:** a School of Public Health, Faculty of Medicine, Imperial College London, London, United Kingdom; b Medical Sciences Division, Nuffield Department of Surgical Sciences, University of Oxford, Oxford, United Kingdom; c Section of Vascular Surgery, Faculty of Medicine, Imperial College London, London, United Kingdom; d Department of Vascular Surgery, Cambridge University Hospitals NHS Foundation Trust, Cambridge, United Kingdom; e Oxford Medical School, Medical Sciences Division, University of Oxford, Oxford, United Kingdom

**Keywords:** Venous insufficiency, Ultrasound, Duplex, Artificial intelligence, Natural language processing, Convolutional neural networks

## Abstract

**Objective:**

To develop and internally validate a prototype multimodal artificial intelligence system for automated Clinical, Etiological, Anatomical, and Pathophysiological (CEAP) classification of venous duplex ultrasound (VDUS) reports, integrating natural language processing of free-text components with computer vision analysis of hand-drawn anatomical diagrams.

**Methods:**

Single-center retrospective observational study using routinely collected clinical data. One thousand consecutive VDUS reports from Cambridge University Hospitals National Health Service Foundation Trust, the United Kingdom (July 2024-May 2025) were labeled according to the CEAP classification, excluding the Etiological component, which could not be reliably determined from duplex reports alone. Transfer learning was applied using ClinicalBERT for text and MobileNetV3 for diagrammatic data. Clinical classes were predicted from request line text. Text- and image-based pathophysiological models were developed for four anatomical territories (great saphenous vein, small saphenous vein, deep system, and perforators), combined using late fusion with probability averaging.

**Results:**

The clinical CEAP model achieved accuracy of 0.91, macro-F1 of 0.82, and macro-area under the curve of 0.98. Pathophysiological prediction varied, with text models broadly outperforming image models. Fusion yielded heterogeneous benefits, improving small saphenous vein performance but reducing deep system accuracy. The performance of the final pathophysiological CEAP fusion models varied across anatomical territories: accuracy ranged from 0.70 to 0.92 and macro-area under the curve from 0.80 to 0.92.

**Conclusions:**

This study demonstrates the feasibility of automated CEAP classification from VDUS reports. Although class imbalance affected minority class predictions, the strong discriminatory performance validates this multimodal machine learning model for extracting clinically meaningful information from real-world data. This approach offers potential, pending external validation, to streamline vascular services through automated triage and guideline-compliant decision-making.

**Article Highlights**

• **Type of Research:** Single-center retrospective observational study
• **Key Findings:** A natural language processing model predicted Clinical, Etiological, Anatomical, and Pathophysiological (CEAP) classes with an accuracy of 0.91 and macro-area under the curve of 0.98. The multimodal fusion models for anatomical-pathophysiological CEAP classification achieved an accuracy of 0.70 to 0.92 and macro-area under the curve of 0.80 to 0.92 across four venous territories.
• **Take Home Message:** A prototype artificial intelligence system integrating natural language processing and computer vision via late fusion can automatically classify three of the four gold standard CEAP criteria from routinely collected venous duplex ultrasound reports, with consistently strong discriminatory performance, offering a proof-of-concept for automated duplex-derived severity scoring.

Chronic venous disease (CVD) of the lower limbs is a globally prevalent condition with significant clinical and economic burden.^1^ The Clinical, Etiological, Anatomical, and Pathophysiological (CEAP) scoring system is the international gold standard used to describe and classify CVD.^2^^,^^3^ CVD becomes the most disabling in its advanced forms, with venous ulcers (C6) highly associated with impaired mobility, psychological distress, and social isolation.^4^ The cost of treating CVD is considerable, with the UK National Health Service (NHS) spending over £2 billion/year managing venous ulcers alone, highlighting the need for more efficient care delivery.^5^^,^^6^

Global CVD care pathways face significant pressures from long waiting lists and rising demand, worsened significantly since the coronavirus disease 2019 pandemic, when venous procedures were consistently deprioritized and often canceled in favor of urgent arterial work.^7^ The critical investigation for patients with CVD is the venous duplex ultrasound (VDUS) scan performed by a specialist vascular scientist, typically reported as a free-text description accompanied by an annotated diagram of the venous tree.^2^ However, the current report format presents significant challenges for research and large-scale analytics: the text lacks standardization, and the hand-drawn diagrams, whereas interpretable by humans, are not readily comparable using traditional computational methods.

Advancements in artificial intelligence (AI), particularly natural language processing (NLP) and machine learning (ML), offer significant potential to enhance the efficiency, consistency, and objectivity of clinical workflows.^8^^,^^9^ In the context of CVD, automated CEAP scoring of VDUS reports would support guideline-compliant decision-making,^2^^,^^4^^,^^10^^,^^11^ enable large-scale analytics on routinely collected vascular data, and facilitate direct comparison between patients – allowing prioritization of those with more severe disease while safely deferring treatment for those with minimal burden. Hence, the development of an automated CEAP scoring technology speaks to the top venous research priority identified by the UK Vascular James Lind Alliance Priority Setting Process, “How can all patients be given the opportunity to access the specialist assessment and treatment they need?,”^6^ by offering a means to increase access to specialist care for those patients who need it most.

## Study objective

The objective of this study was to evaluate whether multimodal AI can be used to automatically classify VDUS reports according to the gold standard CEAP criterion. Herein we detail the conceptualization, development, and internal validation of a prototype AI model, capable of extracting clinically relevant information from both free-text and diagrammatic components of the VDUS reports, producing an automated CEAP score.

## Methods

### Study design and data acquisition

This study used a retrospective observational design using routinely collected clinical data. Reporting follows the Strengthening the Reporting of Observational Studies in Epidemiology statement^12^ and the REporting of studies Conducted using Observational Routinely collected Data extension,^13^ in accordance with the endorsed reporting guidelines of the *Journal of Vascular Surgery: Venous and Lymphatic Disorders.* A completed checklist can be found in the Appendix A1 (online only).

One thousand consecutive VDUS reports were extracted from the vascular laboratory database at Cambridge University Hospitals NHS Foundation Trust (CUH), a large UK tertiary vascular surgery center. This study met the criteria of a service evaluation, assessing the feasibility of automated classification applied to existing routinely collected clinical data, without any change to patient care pathways or prospective intervention. As such, using the “Is my study research?”^14^ and “Do I need NHS Research Ethics Committee review”^15^ decision tools, it was determined that Research Ethics Committee review was not required. Similarly, individual patient informed consent was not required, due to the use of deidentified patient data, with no participants contacted or recruited. The service evaluation was approved locally by the CUH Patient Outcomes Team (Clinical Project ID7165). Data extraction was performed by a clinical team member (H.B.) under consultant supervision (M.G.). The VDUS reports represented a consecutive series extracted directly from the CUH vascular laboratory database, spanning July 23, 2024, to May 28, 2025. The sex of patients could not be ascertained from the duplex reports and is therefore not reported. The sample size of 1000 scans was informed by comparable ML studies in the literature,^16^^,^^17^ and is further justified by the adoption of transfer learning, which substantially reduces data requirements relative to training from scratch.^18^ Each report included: (1) a textual request line provided by the referring clinician, specifying the clinical indication for the scan; (2) an annotated diagram of the venous tree, detailing flow, and any notable pathology; and (3) a textual summary of the scan findings. A sample report is shown further (Fig 1). Images underwent preprocessing consisting of cropping and padding to produce centered square images, with those lacking a detectable anatomical region or exhibiting significant anatomical aberrations, excluded from further analysis.

**Fig 1:**
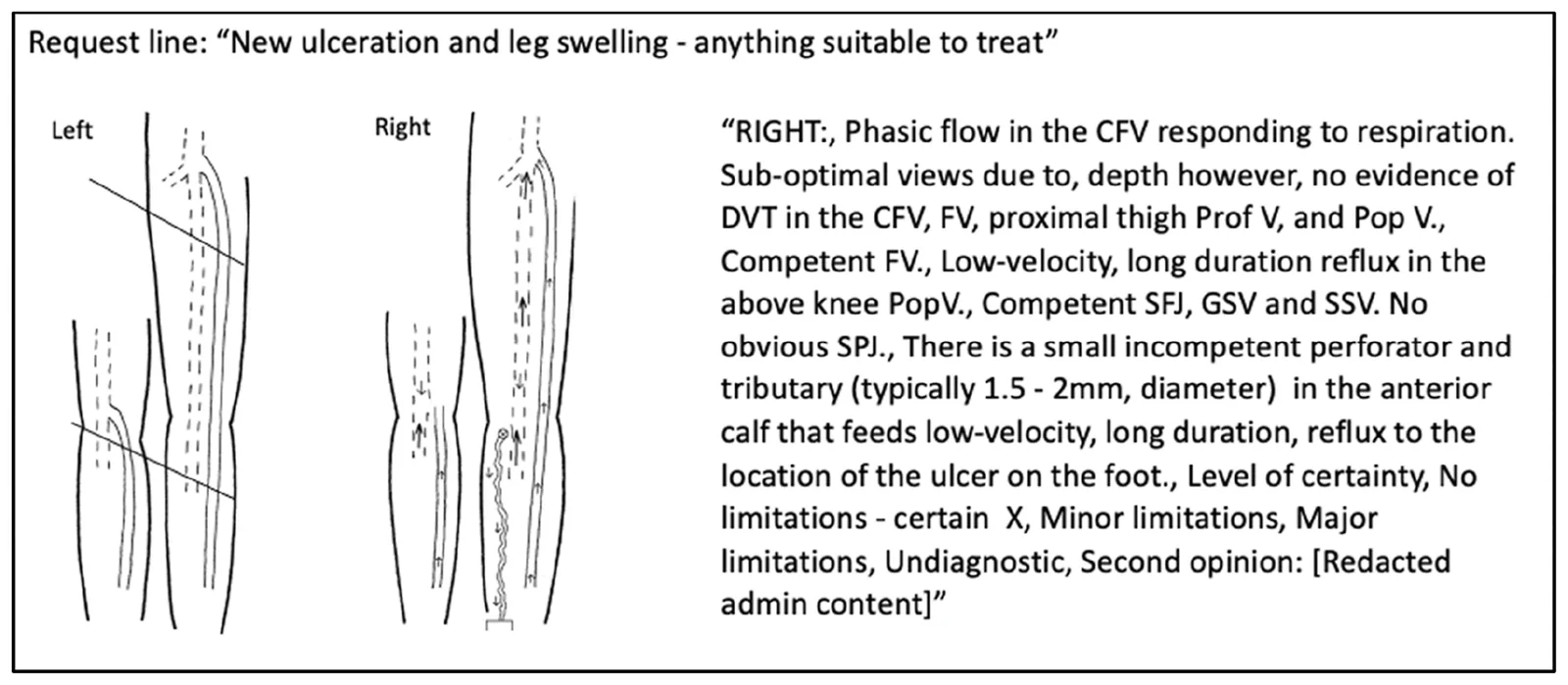
Sample venous duplex report showing the referring clinician’s request line, annotated venous diagram, and corresponding text summary. *CFV*, Common femoral vein; *DVT*, deep vein thrombosis; *FV*, femoral vein; *GSV*, great saphenous vein; *Pop V*, popliteal vein; *Prof V*, profunda vein; *SFJ*, saphenofemoral junction; *SPJ*, saphenopopliteal junction; *SSV*, small saphenous vein.

### Data annotation

The CEAP classification was used to categorize the Clinical, Anatomical, and Pathophysiological status of each report. The Etiological component had to be excluded, as it could not be reliably assessed from the information available in the VDUS reports. Subcategories of the clinical CEAP classes were consolidated to reduce class imbalance; for example, C4a, C4b, and C4c were all recorded as C4. Although this represents a simplification, clinical management is unlikely to differ significantly between subcategories, making this acceptable for prototype development. Regarding the anatomical classification, the basic CEAP anatomical categories of superficial, deep, and perforators were used, with the superficial category further subdivided into great saphenous vein (GSV) and small saphenous vein (SSV). This was possible because the CUH duplex report template included dedicated GSV and SSV regions by default (Fig 1), ensuring sufficient annotated cases for both territories, providing valuable anatomical detail that is highly relevant for treatment planning. The anterior accessory saphenous vein (AASV) was not included as a dedicated region in the template and was therefore rarely annotated. The small number of positive cases, combined with the absence of consistently documented negative cases, precluded reliable modeling of the AASV as a separate venous territory. Clinical CEAP classes were predicted from the unstructured, clinician-entered request line text, which varied considerably in detail and clinical content. Pathophysiological scoring was performed independently for each anatomical territory (GSV, SSV, deep, and perforators), providing both anatomical and pathophysiological CEAP classification. Data labeling was conducted by the lead author (J.C.) and independently validated by a second reviewer (W.J.) across all 1000 reports. Inter-rater reliability was near-perfect across all components: clinical CEAP scoring achieved 98.6% agreement (Krippendorff’s α = .981), text report labeling 98.4% (α = .971), and diagrammatic labeling 98.7% (α = .976).

### Data augmentation and addressing class imbalance

The dataset exhibited substantial class imbalance across clinical and anatomical-pathophysiological domains. The clinical component was evaluated per patient. One patient was excluded as they were the lone C1 patient, with the remaining 999 cases split 80:20 into train and test sets. To address class imbalance in the training set, a three-stage large language model-based augmentation strategy was applied using GPT-4o-mini^19^: synthetic request line generation for the severely under-represented C5 class, rephrasing of all request lines to introduce lexical variation, and final class balancing to 614 records/class [C2-C6 and unable to classify (UC)], yielding a training dataset of 3684 request lines (Fig 2).

**Fig 2:**
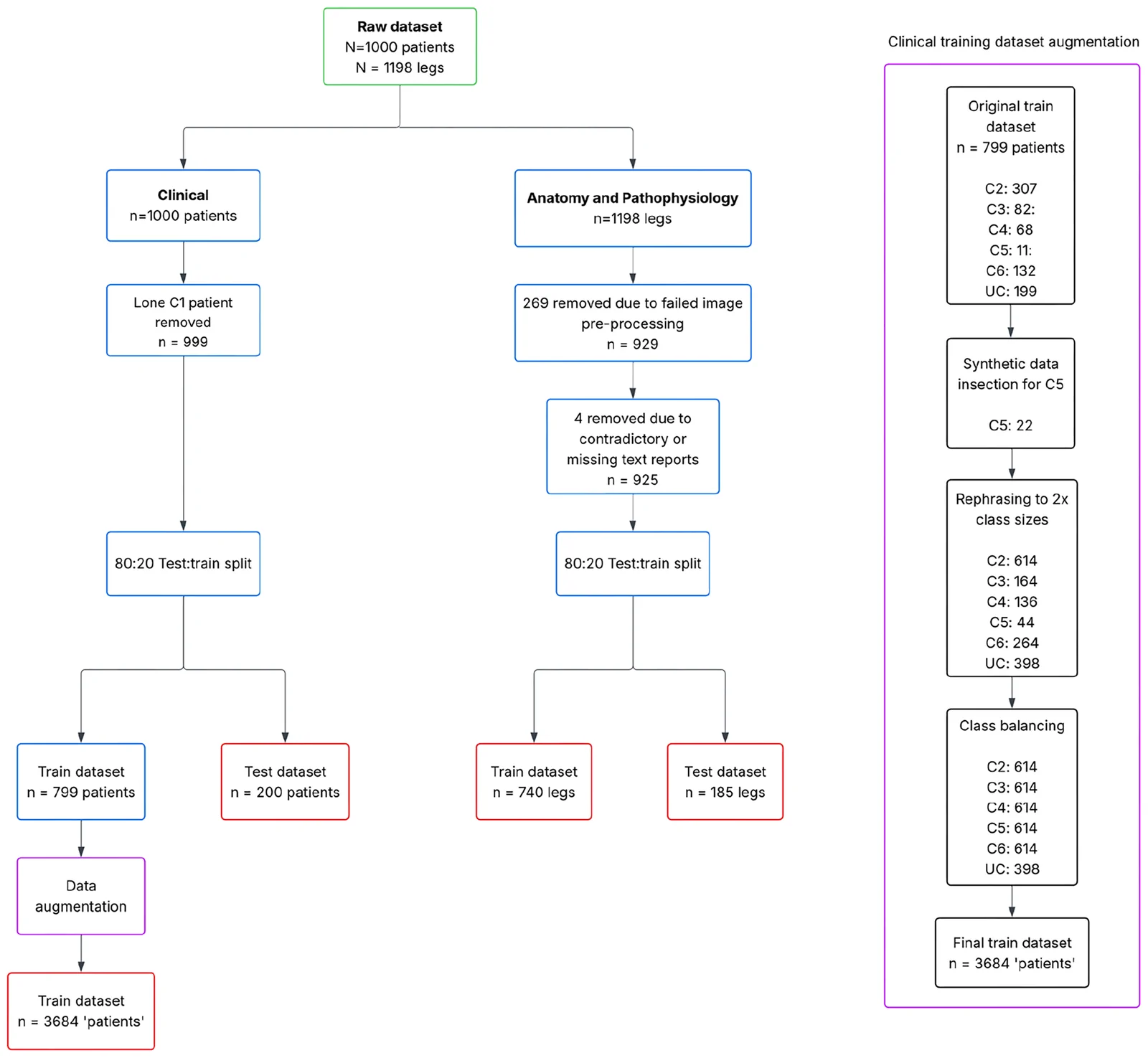
Data preprocessing pipeline for the clinical and anatomical-pathophysiological data. The data augmentation strategy applied to the clinical training dataset, shown in the *purple* box, is expanded on the right. *UC*, Unable to classify.

The anatomical and pathophysiological components were evaluated per leg, with 269 legs failing preprocessing and a further four excluded due to missing or contradictory text summaries, with 925 legs then undergoing an 80:20 split (Fig 2). Image-level augmentation included random rotation (±10°) and brightness and contrast jittering. To address class imbalance, the categories of *obstruction* plus *reflux and obstruction* were merged into a single *obstruction* class to ensure adequate training representation, retaining clinical utility as this combined class represents more severe pathology than reflux alone. A class-weighted loss function was also applied, to ensure the model received proportionally greater feedback from under-represented but clinically meaningful cases.

### Model development

Classification tasks were structured to reflect duplex reporting practice. A single multiclass model predicted clinical CEAP class (C2-C6 and UC) from the request line text. The class *UC* equates to *unable to classify*, reflecting insufficient information on the duplex report to enable categorization, ensuring the model was trained to recognize and predict this class rather than being shielded from ambiguous inputs. For pathophysiological classification, separate models were developed for each venous territory (GSV, SSV, deep, and perforators), each predicting across four classes (*obstruction*, *reflux*, *normal*, and *UC*), except the perforator system, which was restricted to three (*reflux*, *normal*, and *UC*), because of the absence of obstructive cases, consistent with the rarity and limited clinical significance of perforator obstruction on routine duplex imaging.

A transfer learning approach was adopted to leverage pretrained feature extraction capabilities while adapting to the clinical classification tasks. For textual data, ClinicalBERT was used, a transformer-based model pretrained on clinical electronic health records.^20^ For image data, MobileNetV3 (large) was used, a lightweight convolutional neural network originally trained on ImageNet.^21^ In both cases, the pretrained classification head was replaced with a task-specific linear layer. All models underwent progressive layer unfreezing, beginning with the classification head alone before systematically unfreezing deeper layers, preventing catastrophic forgetting of pretrained representations. Hyperparameters were individually optimized for each model via grid search, and all models were trained using the AdamW optimizer with early stopping. For the clinical CEAP model, architecture selection was based on macro-F1, given its balance of precision and recall across classes. For the pathophysiological models, macro-area under the curve (AUC) was instead used, as these models supply probability outputs to the downstream fusion step, and AUC better reflects the quality of ranked probability predictions independent of a fixed classification threshold. A complete list of Python modules and hyperparameter specifications is provided in the Appendix A2 (online only). Late fusion via equal-weighted probability averaging was selected to combine the text and image models for the pathophysiological prediction, offering simplicity and reduced overfitting risk given the modest dataset size. This approach required the establishment of a composite ground truth, established by taking the most severe pathology from either the diagrammatic or textual label, following clinical severity hierarchy (obstruction > reflux > normal > unable to classify). This approach is clinically justified as it ensures that the most significant pathology requiring intervention is captured, reflecting the greatest clinical priority of identifying patients with severe venous disease. Where class imbalance was most severe (SSV and deep territories), percentile-based threshold optimization was applied to the fused probability outputs to improve minority class detection. The fusion model development pipeline is summarized further (Fig 3).

**Fig 3:**
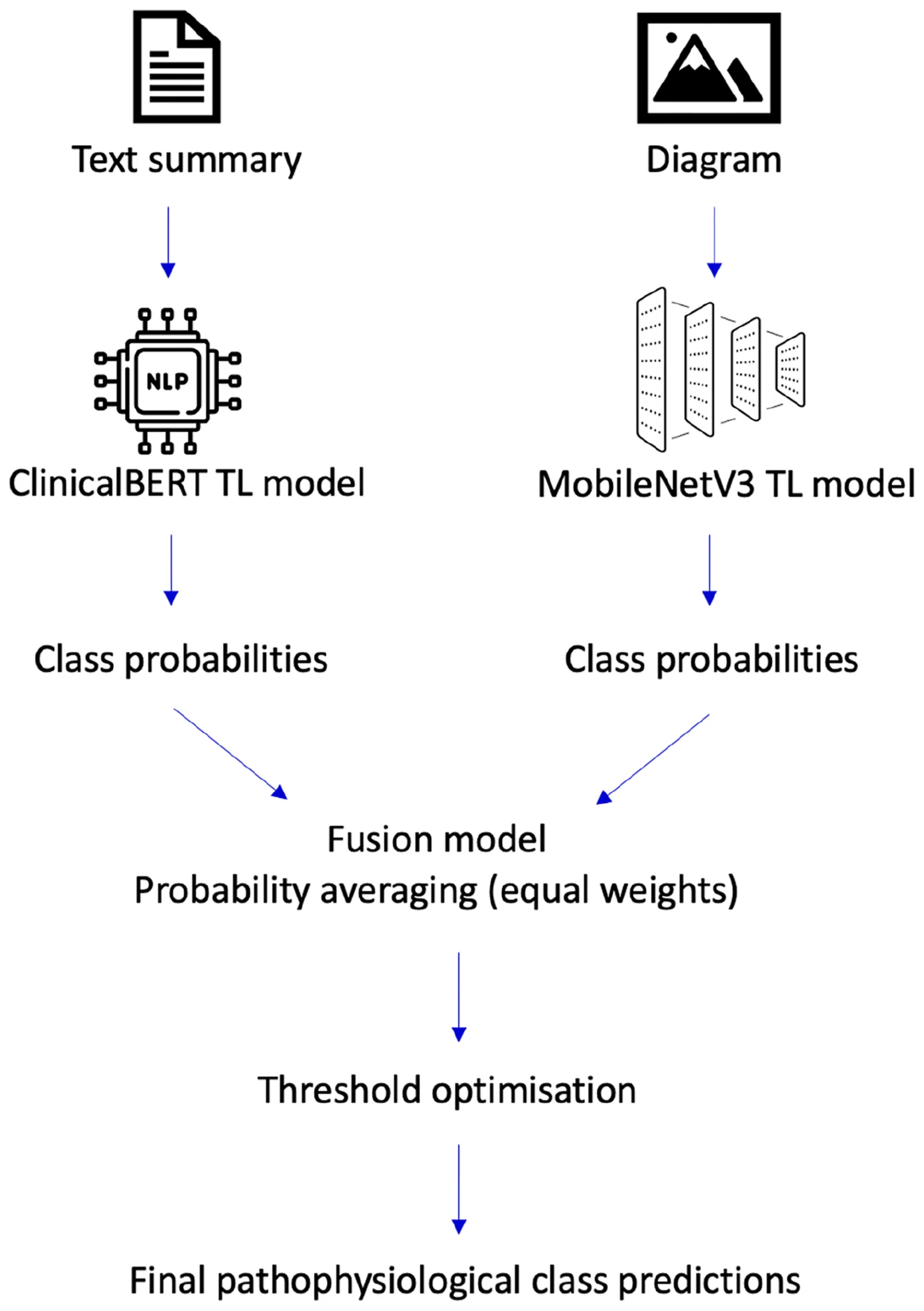
Overview of the model development and fusion pipeline. *TL*, Transfer learning.

### Model evaluation

Models were evaluated on preserved, unaugmented test sets. Macroaveraged metrics were prioritized over weighted averages, as they give equal weight to minority classes regardless of prevalence, although both were calculated. Per-class precision, recall, and F1-score were reported alongside macro- and microaveraged AUC values, with one-vs-rest receiver-operating characteristic (ROC) curves generated for each class. Macro-F1 was the primary evaluation metric, incorporating both precision and recall into a single measure. Confusion matrices were inspected for systematic misclassification patterns. Constituent text and image models were evaluated independently alongside fusion models to assess the marginal contribution of each modality to predictive performance.

## Results

### Clinical CEAP prediction model

The clinical CEAP prediction model achieved strong overall performance: accuracy 0.91, macro-F1 0.82, and macro-AUC 0.98. The ROC curves, classification table, and confusion matrix are shown in Fig 4. The gap between macro- and weighted-F1 (0.82 vs 0.91) was driven primarily by poor performance on the minority C5 class (healed ulcer), which was represented by only three test cases, yielding an F1 0.40 despite an AUC 0.96. All other classes achieved F1 scores 0.88 to 0.94. The most frequent misclassification was six C2 cases predicted as UC, with the model defaulting to the majority class in situations of insufficient information. Training curves are provided in the Appendix A3 (online only).

**Fig 4:**
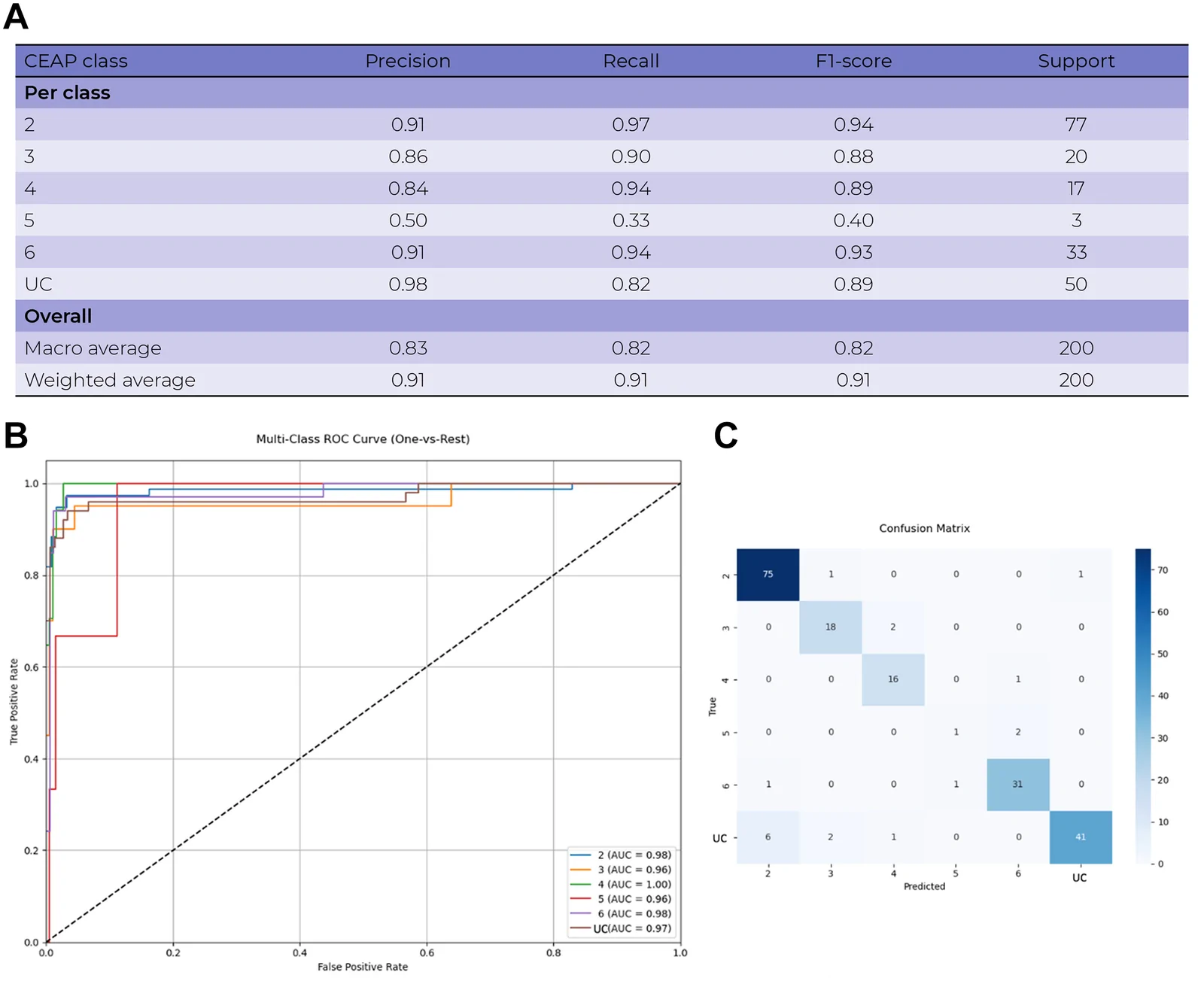
Clinical, Etiological, Anatomical, and Pathophysiological (*CEAP*) prediction model performance. **A**, Per-class classification performance. **B**, One-vs-rest receiver-operating characteristic (ROC) curves with per-class area under the curve (*AUC*) values (macro-AUC, 0.98). **C**, Confusion matrix. *UC*, Unable to classify.

### Anatomical-pathophysiological CEAP prediction models

The classification performance of the four fusion models is summarized in Table I, shown in bold, alongside constituent text and image model results to enable evaluation of each modality’s independent contribution. Training curves and per-class performance for the base models are provided in the Appendix A4 (online only). Per-class performance of the final fusion models is shown in Fig 5, *A*, with confusion matrices and ROC curves in Fig 5, *B* and *C*.

**Table:** Averaged classification performance of text, image, and fusion models across four venous territories

Territories	Modalities	Weighted-precision	Macro-precision	Weighted-recall	Macro-recall	Weighted-F1	Macro-F1	Accuracy	Micro-AUC	Macro-AUC
GSV	Text	0.78	0.63	0.79	0.62	0.78	0.63	0.79	0.93	0.89
	Image	0.57	0.46	0.55	0.45	0.54	0.42	0.55	0.77	0.70
	**Fusion**	**0.77**	**0.65**	**0.77**	**0.62**	**0.77**	**0.63**	**0.77**	**0.90**	**0.85**
SSV	Text	0.62	0.33	0.67	0.33	0.64	0.33	0.67	0.88	0.74
	Image	0.61	0.43	0.64	0.38	0.60	0.39	0.64	0.86	0.69
	**Fusion**	**0.69**	**0.59**	**0.70**	**0.44**	**0.69**	**0.47**	**0.70**	**0.90**	**0.80**
Deep	Text	0.94	0.64	0.96	0.73	0.95	0.68	0.96	1.00	0.99
	Image	0.62	0.36	0.59	0.45	0.61	0.38	0.59	0.86	0.67
	**Fusion**	**0.92**	**0.62**	**0.92**	**0.62**	**0.92**	**0.60**	**0.92**	**0.98**	**0.92**
Perforators	Text	0.84	0.55	0.86	0.53	0.85	0.54	0.86	0.94	0.84
	Image	0.54	0.34	0.56	0.35	0.55	0.35	0.56	0.79	0.49
	**Fusion**	**0.85**	**0.56**	**0.86**	**0.53**	**0.85**	**0.54**	**0.86**	**0.94**	**0.80**

*AUC*, Area under the curve; *GSV*, great saphenous vein; *SSV*, small saphenous vein; *UC*, unable to classify.

Fusion model results are shown in bold. Weighted-precision, weighted-recall, and weighted-F1 are weighted by class support; macro-precision, macro-recall (macro-F1), and macro-AUC give equal weight to all classes regardless of prevalence.

**Fig 5:**
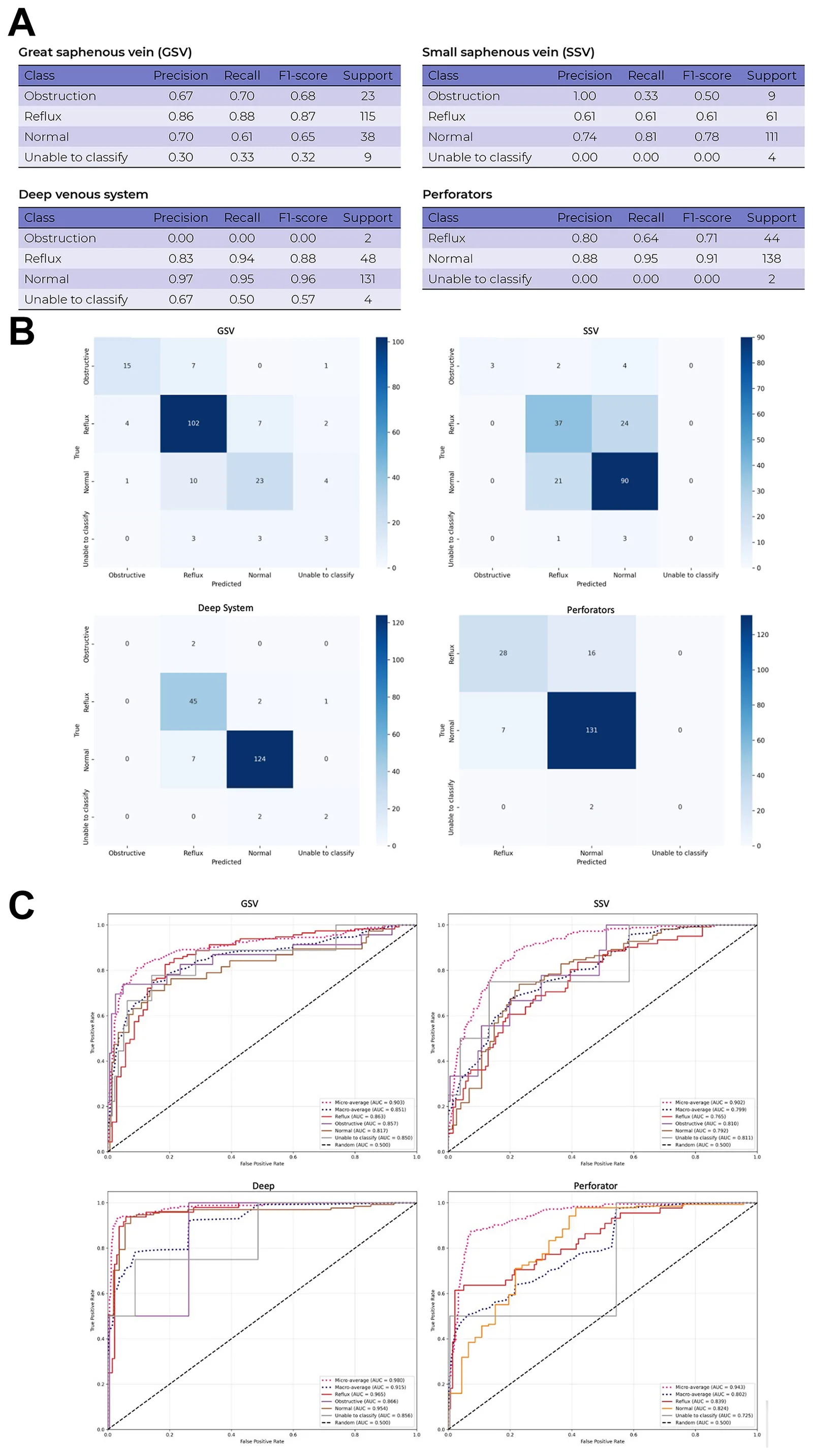
Per-class performance of the final four fusion models. **A**, Per-class classification performance. **B**, Confusion matrix. **C**, One-vs-rest receiver-operating characteristic (ROC) curves for each fusion model displaying per-class area under the curve (*AUC*) values (*solid lines*), with macro- and microaveraged AUC (*dotted lines*). *GSV*, Great saphenous vein; *SSV*, small saphenous vein.

Fusion model performance varied across venous territories: accuracy ranged from 0.70 to 0.92 and macro-F1 from 0.47 to 0.63. Discriminatory performance was consistently stronger, with macro-AUC ranging from 0.80 to 0.92. Text models outperformed image models across all territories except SSV. Fusion yielded heterogeneous benefits: the SSV territory showed clear improvement over both constituent models, whereas the deep territory showed modest deterioration from its strong text baseline. Macroaveraged metrics were consistently depressed relative to weighted averages, driven by poor performance on minority classes, particularly *obstruction* and *unable to classify*.

The dominant misclassification pattern across all territories was confusion between *reflux* and *normal*, which is consistent with the ordinal structure of the classification scheme. This was most pronounced in the SSV territory, where 39% of true *reflux* cases were predicted as *normal*. By contrast, misclassifications between nonadjacent categories were rare across all models.

#### Great saphenous vein

The GSV fusion model achieved macro-F1 0.63 and accuracy 0.77. *Reflux* was most reliably identified (F1, 0.87; AUC, 0.86), and *obstruction* was stable (F1, 0.68; AUC, 0.86). By contrast, *normal* vein detection declined (F1, 0.65; AUC, 0.81; F1, −0.08; AUC, −0.11) and *unable to classify* remained weak despite strong discriminatory performance (F1, 0.32; AUC, 0.85).

#### Small saphenous vein

The SSV fusion model achieved the largest gains over its constituent models: macro-F1 0.47 and accuracy 0.70, representing improvements of +0.14 and +0.03 over the text model respectively. *Reflux* (F1, 0.61; AUC, 0.77) and *normal* (F1, 0.78; AUC, 0.79) and *obstruction* (F1, 0.50; AUC, 0.81) metrics all improved vs baseline model performance. *Unable to classify* was undetected (F1, 0.00; AUC, 0.81).

#### Deep system

The deep fusion model achieved the highest accuracy (0.92), with strong performance for *normal* (F1, 0.96; AUC, 0.85) and *reflux* (F1, 0.88; AUC, 0.97). However, macro-F1 declined by 0.08 relative to the baseline text model, driven by deterioration in *unable to classify* (F1, 0.57; AUC, 0.86; F1, −0.23; AUC, −0.14) and persistent failure to detect *obstruction* (F1, 0.00; AUC, 0.87).

#### Perforators

The *perforator fusion model* preserved text-level performance (macro-F1, 0.54; accuracy, 0.86) without clear added value from fusion. *Normal* veins were reliably detected (F1, 0.91; AUC, 0.82), whereas *reflux* showed modest improvement over text (F1, 0.71; AUC, 0.89; F1, +0.01; AUC, +0.03). *Unable to classify* remained poorly detected in the test set (F1, 0.00; AUC, 0.73).

## Discussion

This is the first study to integrate NLP and computer vision for automated venous duplex severity classification, predicting three of the four elements of the gold standard CEAP classification. Three principal findings emerged: (1) clinical CEAP categories can be accurately predicted from sparse referral text, (2) pathophysiological CEAP prediction is challenging, with text models consistently outperforming image-based models, and (3) multimodal fusion provides heterogeneous benefits across venous territories but demonstrates consistently strong discriminatory performance.

### Clinical CEAP prediction

The clinical CEAP prediction model achieved high accuracy (0.91) and strong macro-F1 (0.82), demonstrating that NLP can effectively extract disease severity information from unstructured referral text – a data source generally considered of poor clinical value. The gap between macro- and weighted-F1 was driven primarily by the poor performance of the minority C5 class (n = 3 test cases; F1, 0.40), despite strong discriminatory performance (AUC, 0.96).

### Anatomical-pathophysiological CEAP prediction

Text models outperformed image models across all venous territories except SSV. This likely reflects the enhanced domain-specific pretraining of ClinicalBERT relative to MobileNetV3, the greater standardization of clinical text compared with hand-drawn diagrams, and the more direct encoding of pathophysiological states in text vs the complex spatial interpretation required for diagrammatic data. Image model performance was particularly limited for perforators, where the lack of spatial consistency in diagram annotation likely prevented the model from learning meaningful spatial patterns. Class imbalance was the principal source of performance distortion across pathophysiological models, most clearly illustrated by the deep text model, which achieved an AUC of 0.99 but F1 of 0.00 for *obstruction*, reflecting meaningful learned representations that were insufficient for categorical prediction across only very few test cases.

Fusion yielded heterogeneous results, improving performance in the SSV territory while causing modest deterioration in the deep system. This reflects established principles of multimodal AI: fusion adds noise when one modality already achieves near-optimal performance, as seen with the deep text model, where standardized and unambiguous reporting of deep venous pathology left little complementary information for the image modality to contribute. Conversely, fusion is most valuable when modalities provide complementary information, as in the SSV territory. These findings highlight that the most complex solution is not always optimal.

### Contextualizing results

A recent comprehensive systematic review encompassing 212 vascular ML studies reported a median AUC of 0.88.^22^ The clinical CEAP prediction model confidently surpassed this benchmark, with both micro- and macro-AUCs of 0.98. All fusion models exceeded this benchmark for micro-AUC (range, 0.90-0.98), with the deep fusion model also surpassing it for macro-AUC. Compared with similar models in the literature, the clinical CEAP prediction model outperformed Barulina et al^23^ (macro-F1, 0.79) but did not reach the performance of Denisov et al,^24^ (macro-F1, 0.84 – calculated from per-class results) or Oliveira et al^16^ (macro-F1, 0.96). However, the latter two papers used simplified classifications, with the first using just C0 to C2, and the second collapsing with C5 and C6 into a single class. Comparison is also limited by differences in input modality: photo-based systems such as these evaluate patients’ physical signs directly, whereas this model processes clinician-generated reports and therefore inherits the variability of human interpretation. Notably, the inter-rater agreement for clinical CEAP classification has recently been reported at 63%.^25^ Hence, the value of this system lies not in surpassing the accuracy of human assessment, but in providing consistent, scalable processing of existing clinical documentation – reflecting the growing consensus that AI is best deployed to augment rather than replace clinical judgment.^26^

### Strengths and limitations

This study has several notable strengths. The integration of multimodal duplex data for CEAP classification is genuinely novel, moving beyond existing photo-based approaches that solely evaluate the clinical CEAP class, to produce a model with utility across key areas such as triage and guideline-compliant care. Inter-rater reliability was assessed across all 1000 reports, considerably more rigorous than the subset validation typically reported, with near-perfect agreement across all labeling components (Krippendorff's α > .97). The use of real-world NHS data ensures the model learns features from authentic vascular surgery patients, unlike studies relying on web-scraped datasets of uncertain origin and quality. Finally, this study reports on a diverse array of evaluation metrics, including class-level reporting, which is essential in imbalanced clinical datasets.

There were several limitations to this study, which can be grouped into data and algorithmic elements. A key data limitation is the single-center design, which constrains generalizability across institutions with different reporting conventions, consistent with this study’s classification as a service evaluation. Institutional performance findings are not intended to generalize beyond CUH, though the underlying multimodal methodology may be transferable pending external validation. The dataset size, while sufficient for prototype development, increases overfitting risk and limited classification performance estimation for minority classes. The use of routinely collected data introduces potential misclassification bias, as reporting style and completeness may have varied between vascular scientists given the absence of a standardized protocol. However, this real-world variability is precisely the operational context for which this system is designed. The dataset structure necessitated pragmatic simplification of the 2020 update of CEAP, including consolidation of clinical subcategories and exclusion of the AASV as a distinct anatomical territory. Future work would benefit from duplex report templates standardized in line with current CEAP guidance, particularly for the superficial venous system, enabling more complete automated classification across venous territories. Composite ground truth construction for fusion model evaluation introduced a further limitation, with label conflicts resolved by clinical severity hierarchy, which had the potential to produce an evaluation task not aligned with either modality’s original training objective. However, discarding conflicts would have reduced dataset size and introduced selection bias, making hierarchical resolution necessary to preserve comprehensiveness while safeguarding clinical priorities. Indeed, conflicting labels between modalities in real-world clinical data remains a largely unaddressed challenge in the multimodal AI literature.^27^^,^^28^ Regarding the algorithms used, MobileNetV3 was pretrained exclusively on photographic images, with no exposure to medical data or hand-drawn diagrams. The inherent difficulties of interpreting hand-drawn medical diagrams – with variable line widths, noise, and ambiguous arrowhead-to-structure linkage – represents a fundamental challenge likely not fully resolved by the approaches used. However, the reality is that no open-source computer vision algorithms for medical diagram exist, and MobileNetV3’s efficient architecture and customization options made it best suited for this task.

### Clinical implications and future directions

This technology could operate at the point of VDUS report generation, producing a standardized CEAP score that could be appended to the report in the electronic patient record. This would support guideline-compliant decision-making – for example, in the United Kingdom, the National Institute of Health and Care Excellence recommend compression hosiery for C4 to C6 disease, and only for C2 disease if interventional treatment is unsuitable.^4^^,^^10^^,^^11^ Standardized CEAP scoring also enables direct comparison between patients, supporting more effective triage – similar benefits have been demonstrated in emergency department, where AI-assisted triage provided safety and efficiency gains.^29^^,^^30^ More broadly, the model could help address documented health equity gaps in venous disease management. In England, it has been shown that non-White ethnicities receive 40% to 60% fewer procedures despite similar or higher disease burden, and that socioeconomic disparities result in 20% to 30% fewer procedures among younger patients in less-deprived quintiles.^31^ One contributing mechanism may be variability in how disease severity is documented at referral, which automated CEAP scoring could address by grounding triage decisions in objective duplex-derived severity rather than variable referral quality, supporting more equitable treatment decisions. Beyond this, duplex-derived CEAP scores could even serve as inputs for downstream ML models, such as predicting venous ulcer healing. Most existing models rely primarily on wound morphology,^32^ but incorporating CEAP scores would capture the underlying venous pathoanatomical state, and would likely add substantial predictive value.

## Conclusions

This study represents the first successful integration of NLP and ML for automated venous duplex severity classification. The clinical CEAP prediction model demonstrated strong discriminatory performance (macro-AUC, 0.98) and robust classification performance (accuracy, 0.91; macro-F1, 0.82). Anatomical-pathophysiological CEAP prediction was more challenging, with text models outperforming image-based approaches, likely due to standardized clinical reporting vs variable hand-drawn diagrams. Multimodal fusion yielded heterogeneous results, providing substantial improvements in some territories while offering no benefit in others. However, all fusion models demonstrated strong discriminatory performance (macro-AUC, 0.80-0.92). Thus, this work provides a proof-of-concept for automated CEAP classification from venous duplex reports. Arguably, the most significant impact of this work lies in enabling duplex scan data to be processed by ML models for the first time, unlocking a previously inaccessible source of structured clinical intelligence for downstream applications. Taken together, these findings demonstrate the feasibility of harnessing venous duplex data with AI, highlighting a direction that, pending external validation, could support more advanced and efficient vascular care.

## Author Contributions

Conception and design: JC, HB, AD

Analysis and interpretation: JC, HB, WJ, AD

Data collection: JC, HB, WJ, MG

Writing the article: JC, WJ, AD

Critical revision of the article: JC, HB, WJ, MG, AD

Final approval of the article: JC, HB, WJ, MG, AD

Statistical analysis: JC

Obtained funding: Not applicable

Overall responsibility: JC

## Funding

This study received no specific grant funding from any funding agency in the public, commercial, or not-for-profit sectors. The lead author’s MSc program, from which this work originates, was supported by the School of Public Health Scholarship at 10.13039/501100000761Imperial College London.

## Disclosures

The authors have no competing interests.

## Data and code availability

The dataset comprises routinely collected clinical data and cannot be shared publicly due to data governance restrictions, but may be available upon reasonable request subject to appropriate approvals. The Python code used for model development and evaluation is publicly available at: https://github.com/JoeCUK/automated-CEAP-multimodal/.

## Declaration of generative AI and AI-assisted technologies in the writing process

During the preparation of this work, the lead author used Claude Sonnet 4.6 (Anthropic) to assist with development and debugging of the analysis code, prepare the code repository for public sharing, and improve readability and assist with manuscript editing. All AI-assisted code was reviewed, tested, and validated by the lead author, and all manuscript content was reviewed and edited before submission. The lead author takes full responsibility for the content of the publication.

## Appendix (online only)

Appendix A1

Appendix A2

Appendix A3

Appendix A4
