# Supplementary material for: Automated CEAP classification of venous duplex reports using multimodal artificial intelligence
Source: J Vasc Surg Venous Lymphat Disord. 2026 Sep 1;15(1):102613. doi: 10.1016/j.jvsv.2026.102613 (PMC13675709; doi:10.1016/j.jvsv.2026.102613)
Supplement: Supplementary file 2 [file mmc2.docx]

| **Module** | **Version** |
| --- | --- |
|  |  |
| datasets | 3.6.0 |
| matplotlib | 3.9.4 |
| numpy | 1.23.5 |
| openai | 1.83.0 |
| pandas | 2.2.3 |
| scikit-learn | 1.6.1 |
| seaborn | 0.13.2 |
| torch | 2.7.1 |
| torchvision | 0.22.1 |
| tqdm | 4.67.1 |

A2: External modules and corresponding versions employed during all stages of data processing, implemented in Python v3.9.6.
