# Supplementary material for: Automated CEAP classification of venous duplex reports using multimodal artificial intelligence
Source: J Vasc Surg Venous Lymphat Disord. 2026 Sep 1;15(1):102613. doi: 10.1016/j.jvsv.2026.102613 (PMC13675709; doi:10.1016/j.jvsv.2026.102613)
Supplement: Supplementary file 4 [file mmc4.docx]

A4: Training and validation curves (loss and validation macro-AUC) for the text and image models
